# Plastic bronchitis due to adenoviral infection: a case report

**DOI:** 10.1186/s12887-020-1954-0

**Published:** 2020-02-10

**Authors:** Fei Zhou Zhang, Lu Qin, Jie Xin Yuan, Lan Fang Tang

**Affiliations:** Zhejiang University School of Medicine of Children’s Hospital, 3333 Binsheng Road, Hangzhou, 310051 China

**Keywords:** Plastic bronchitis, *Human adenovirus 7*, Bronchoscopy, Extracorporeal membrane oxygenation

## Abstract

**Background:**

Plastic bronchitis (PB) frequently occurs as a serious postoperative complication of the Fontan procedure. The definitive causes of PB are unknown.

**Case presentation:**

Herein, we report a pediatric case of PB secondary to adenoviral infection. A 4-year-old girl was admitted to the general pediatric ward for cough since 2 weeks and fever since 11 days. Consolidated lesions were noted in the right upper and both lower lung lobes. Extracorporeal membrane oxygenation was performed because the patient’s respiratory failure remained unalleviated despite the use of a ventilator. Bronchial dendritic casts were extracted using flexible bronchoscopy, and the patient’s breathing improved. Pathological examination of the dendritic cast confirmed the diagnosis of type I PB. The exfoliated cells of sputum and cells from bronchoalveolar lavage fluid were positive for adenoviral antigen*. Human adenovirus 7* was detected by next-generation sequencing of the bronchoalveolar lavage fluid. The patient recovered and was discharged 39 days after admission without recurrence of cough or wheezing.

**Conclusions:**

PB due to *human adenovirus 7* infection should be considered in children with persistent respiratory failure. Flexible bronchoscopy should be performed early to confirm diagnosis and to remove any airway obstruction.

## Background

Plastic bronchitis (PB), which is characterized by the formation of endobronchial casts of undefined etiology, results in confined or extensive bronchial blockage, ventilation defects, dysfunctional gas transfer, and even life-threatening respiratory and circulatory failure [1]. Cases of PB are divided into the following two types: type I (inflammatory casts) predominantly involves fibrin with dense eosinophilic infiltration and is usually the underlying cause of inflammatory lung disease, and type II (acellular casts) predominantly involves mucin, without any acute inflammatory infiltration and occasional appearance of few mononuclear cells [2]. Pediatric cases of PB have been reported in several diseases, such as asthma, allergic diseases, cystic fibrosis, and acute chest syndrome associated with sickle-cell disease [3]. Current studies focus on Fontan treatment for the therapeutic management of complicated cases of childhood congenital heart diseases-induced PB, which is an important complication with a mortality rate of approximately 4–14% [4]. *Human adenovirus 7*, an important member of the human adenovirus family, a main cause of acute respiratory tract infection, especially among the Asian population, including that in our area, can cause pharyngeal conjunctival fever, acute encephalitis, and even fatal pneumonia [5]. To our knowledge, pediatric cases of PB secondary to adenovirus 7 infection have not been reported. Herein, we report the case of a child with *human adenovirus 7* infection associated with fatal PB, and highlight the clinical features, diagnosis, and therapy for this rare condition.

## Case presentation

A 4-year-old girl was referred to the general pediatric ward because of intractable hypoxemia, cough since 2 weeks, and fever since 11 days. Her highest body temperature was 40.6 °C. Peripheral blood cell counts were within the normal range, and etiological examination was not performed due to technical limitations. Azithromycin, ceftriaxone, cefoperazone sodium, and sulbactam sodium were previously administrated in the given order at a local hospital for infection. In the emergency department, face-mask ventilation was administered to maintain the blood oxygen saturation level over 95%. The patient is the second child of non-consanguineous parents, and with an uneventful birth and medical history. On physical examination, coarse breath sounds and wet rales were observed in both lungs. The suprasternal and supraclavicular fossa showed obvious depression.

Laboratory examinations on admission showed increased white blood cell count 21.33 × 10^9^ cells/L (normal range, 4.0–12.0 × 10^9^/L) with 91.4% neutrophils, hemoglobin level of 121 g/L (normal range, 110–155 g/L), platelets 258 × 10^12^/L (normal range, 100–400 × 10^9^/L), hypersensitive C-reactive protein level of 44.97 mg/L (normal range, < 8 mg/L), and procalcitonin level of 25.73 ng/mL (normal range, < 0.46 ng/mL). Biochemical examination revealed reduction in albumin (normal range, 32.0–52.0 g/L), and increase in alanine aminotransferase (103 U/L, normal range, < 50 U/L) and aspartate aminotransferase levels (216 U/L, normal range, 15–60 U/L). Immunofluorescence of exfoliated cells of sputum for adenoviral, respiratory syncytial virus, influenza virus, and parainfluenza virus were weakly positive for the adenoviral antigen*.* RNA of *Mycoplasma pneumoniae* in sputum, antibodies for *Mycoplasma pneumoniae* and *Chlamydia pneumoniae* in blood, and 16S rRNA screening for gram-positive and gram-negative bacteria, and multiple blood cultures (e.g., L-form bacteria, anaerobic bacteria, fungus) were negative. The results of tests related to the tuberculosis antibody—enzyme-linked immunospot, tuberculin, (1, 3) B-D glucan, and Galactomannan were negative.

**Fig. 1:**
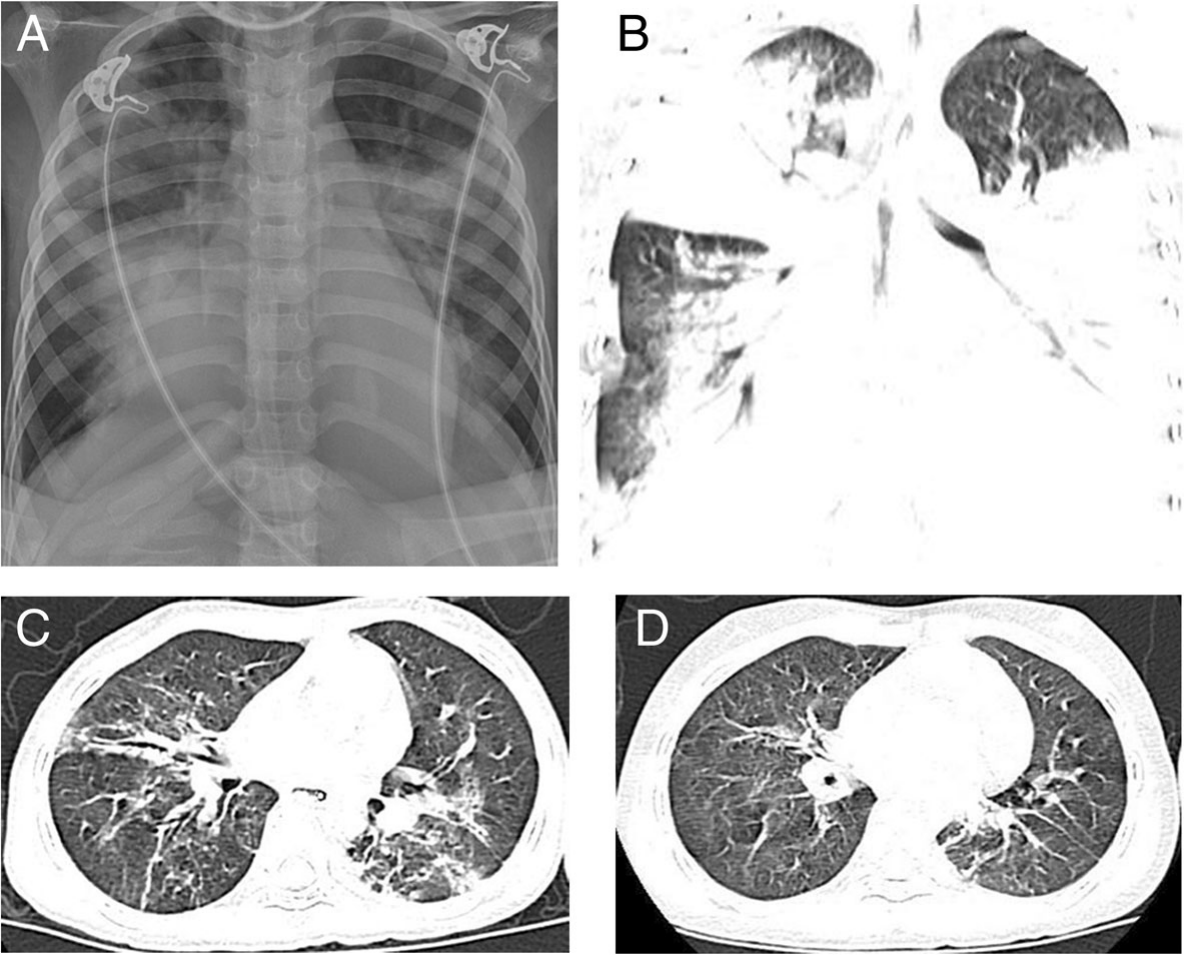
Radiology findings. **a** Chest radiography scan shows consolidation in the right upper and both lower lung lobes; **b** Chest computed tomography (CT) scan shows consolidation in the right upper and both lower lung lobes; **c** Chest CT scan shows significant resorption of the consolidated lesions; **d** Chest CT scan shows an almost complete disappearance of the consolidated lesions

Chest radiography and computed tomography (CT) scans indicated features of consolidation and progressive worsening, especially in the right upper and both lower lung lobes. Signs of airway obstruction (e.g., finger-in-glove pattern or bronchiectasis disappearance) were not obvious. (Fig. 1a-b). Ultrasound examination showed bilateral pleural effusion of approximately 0.77 cm and 1.74 cm thickness in the left and right sides, respectively. Additionally, peritoneal (maximum depth 5.0 cm) and pelvic (maximum depth 2.1 cm) effusions were noted. Thoracentesis was performed 6 days after admission, and approximately 50 mL of slightly turbid, yellowish pleural effusion fluid was collected.

**Fig. 2:**
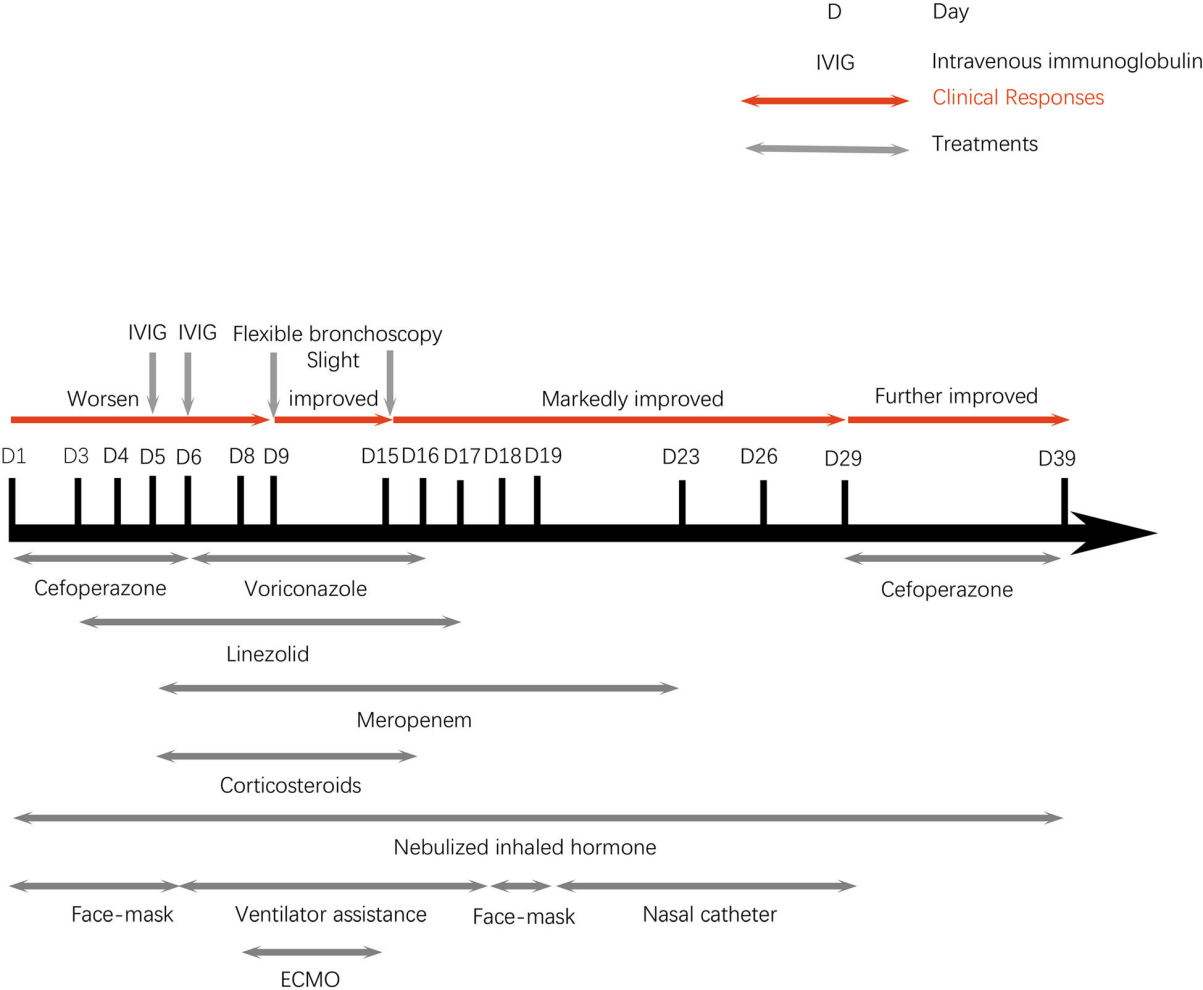
Diagrammatic representation of the treatment and outcome

**Fig. 3:**
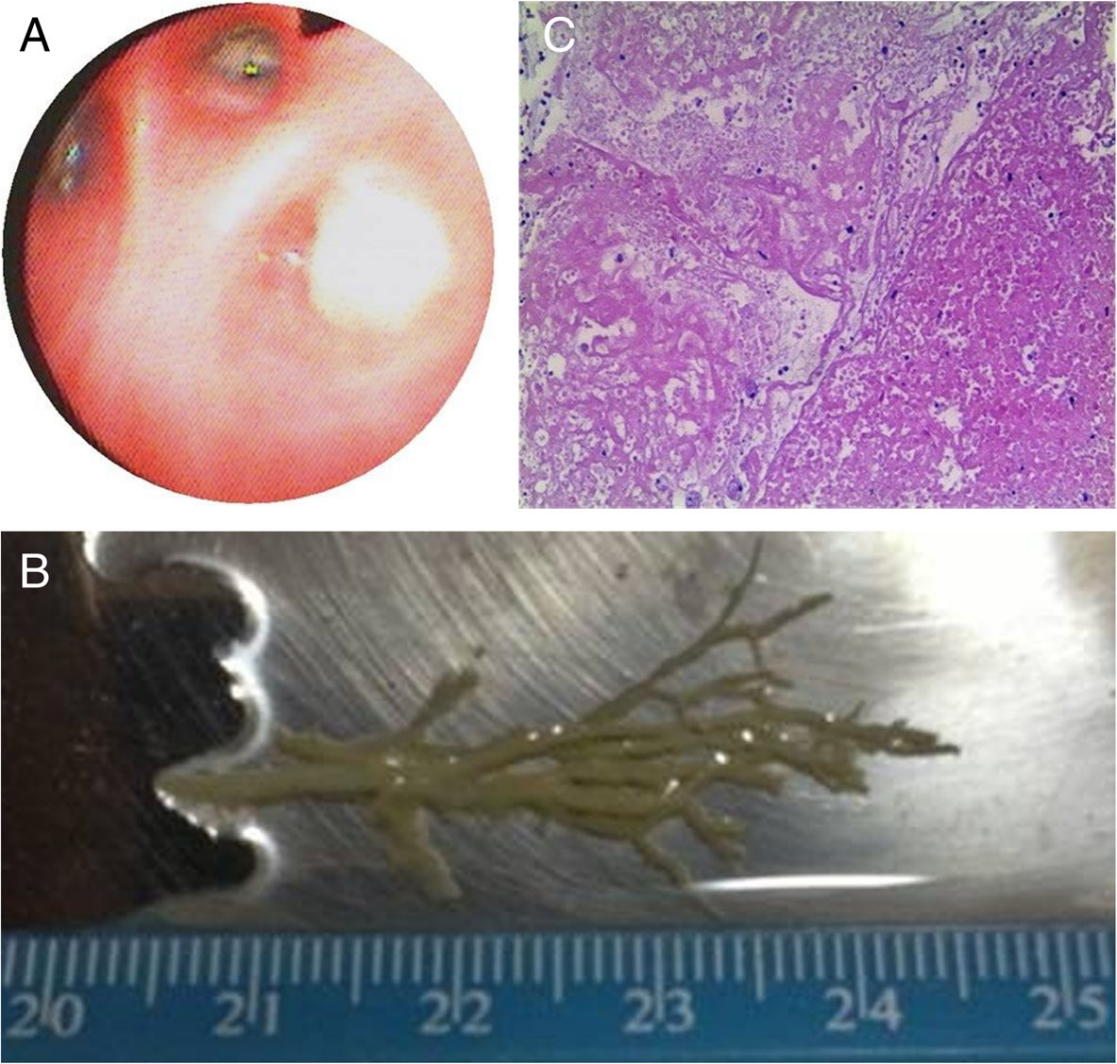
Findings of bronchoscopic and histopathological examinations. **a** Flexible bronchoscopy showed complete obstruction of the right bronchiole by a white and elastic substance. **b** A bronchial tree-like plastic dendritic cast was extracted using flexible bronchoscopy. **c** Hematoxylin-eosin staining of the bronchial tree-like casts shows flaky cellulose-like necrotic tissue with extensive eosinophilic infiltration (× 100)

Although antibiotics such as sulperazone, meropenem, voriconazole, and linezolid; corticosteroids; inhaled oxygen; and additional supportive treatments such as parenteral nutrition and external cooling were administered, the patient experienced type II respiratory failure with face-mask oxygen inhalation (Fig. 2). Her white blood cell count decreased to 1.45 × 10^9^/L with a neutrophil count of 0.87 × 10^9^/L. Anemia (hemoglobin, 86 g/L) and thrombocytopenia (platelets, 78 × 10^9^/L) were noted 5 days after admission. Bone marrow examination showed significant granulocyte proliferation, consistent with infection. Mechanical ventilation was adopted. However, the patient’s respiratory failure did not alleviate, and airway obstruction was speculated. Extracorporeal membrane oxygenation (ECMO) was implemented on the 7th day after admission, and flexible bronchoscopy was performed on the 8th day after admission. Flexible bronchoscopy showed complete obstruction of the right bronchiole by a white and elastic substance (Fig. 3a). Tree-like plastic casts were removed from the bronchi (Fig. 3b). Pathological examination showed flaky and cellulose-like necrotic tissue with extensive eosinophil infiltration consistent with type I PB (Fig. 3c). Immunofluorescence of bronchoalveolar lavage fluid (BALF) cells for respiratory viruses was positive for the adenoviral antigen and negative for respiratory syncytial virus, influenza virus and parainfluenza virus. Pathogen analysis of BALF was performed by next-generation sequencing (BGISEQ-50), and the data were compared with pathogen sequences deposited in the 4 Microbial Genome Databases, which included 3446 species of bacteria, 206 species of fungi, 4152 species of viruses, and 140 species of parasites. The amplified sequences showed a 99.80% match with that of the *human adenovirus 7*. BALF and pleural effusion cultures were negative.

Type II respiratory failure improved after the plastic casts were successfully removed. ECMO support lasted for 7 days, and a subsequent flexible bronchoscopy performed 15 days after admission showed no recurrence of bronchial tree-like plastic casts. Chest radiography and CT showed gradual resorption of the pulmonary consolidation (Fig. 1c), and eventually, ventilator support was removed. Nebulized inhaled corticosteroid was administered on all 39 days; methylprednisolone was administered for 11 days to inhibit systemic inflammation. Antibiotics (meropenem, voriconazole, linezolid, and cefoperazone), intravenous immunoglobulin (IVIG), red blood cells and plasma, albumin infusion, and parenteral nutrition were additionally administered (Fig. 2). The patient was discharged 39 days after admission and continued to receive nebulized inhaled hormone therapy for 7 days. During the subsequent one-year follow-up, chest CT demonstrated improvement of obstructive features (emphysema or atelectasis) (Fig. 1d), with no complaints of recurrent cough or wheezing.

## Discussion and conclusion

PB is an acute and critical pediatric condition, which causes extremely severe cardiopulmonary failure. Histopathology examination of specimens from our patient revealed patchy, fibrinoid, necrotic tissue with extensive acute and chronic inflammatory cell infiltration, and eventually, a diagnosis of type I PB was rendered. Hence, in patients with irreversible respiratory failure, PB should be considered as a differential diagnosis. CT scan may yield a finger-in-glove pattern in case of widespread plugging of the central airways. In this case, there was no central airway blockage, and only suggested signs of atelectasis were observed.

Accurate epidemiological data of this rare condition are still lacking. Most cases have been reported in children with congenital cardiac defects. Recently, new etiologies of PB have been identified, such as human *bocavirus*, *influenza virus*, and *adenovirus* B1 infections; tumor infiltration; and lung transplantation [3, 6–12]. To the best of our knowledge, this is the first case of *human adenovirus* 7 infection-induced PB. Adenovirus-induced pneumonia is a common virus-induced pneumonia in China, characterized by acute fever, radiological features of consolidation, and ineffective outcomes with antibiotic therapy [13]. As human cells are permissive to the growth and replication of human adenoviruses, the virus replicates in the airway epithelium, inducing toxic inflammatory storm (similar to that in our case), and eventually, cell lysis and cell death. Serious cases of human adenovirus infection present with exfoliation of epithelium, infiltration of inflammatory cells and mucus in the bronchial cavity, and occlusion of the bronchial lumen (obstructive bronchitis). This may have been the risk factor for PB in our case. Moreover, direct infection, inflammatory storm, hypoxemia, and carbon dioxide retention may impair the functions of other important organs (e.g., liver) [14]. Hence, *human adenovirus 7* should be investigated as a causative pathogen in patients with PB.

The clinical manifestations of PB are diverse; the main features include cough, chest pain, wheezing, shortness of breath, severe respiratory distress, severe systemic hypoxia, and death due to cardiopulmonary failure and multiple organ dysfunction [15]. Besides cough, persistent high fever, damage to liver functions, and multi-serous cavity effusion were observed in our patient with type II respiratory failure, which remained unalleviated despite ventilator support and necessitated ECMO support.

The gold standard for the diagnosis of PB is the detection of bronchial dendritic casts with flexible bronchoscopy [16]. To date, there is no effective anti-adenovirus treatment. IVIG and corticosteroids were suggested for severe adenovirus pneumonia. Removal of the airway-occluding plastic casts using flexible bronchoscopy and bronchoalveolar lavage was most vital for treating PB [17]. Recently, other treatments of PB have mainly focused on percutaneous thoracic intervention after the Fontan procedure, including the administration of low-dose azithromycin, inhaled bronchodilators, and use of recombinant human DNase [18–20]. In addition, nebulization with N-acetylcysteine and biphasic cuirass ventilation have also been reported [21]. Similarly, in our case, hypoxemia was resolved after removal of the plastic casts. ECMO aided patient recovery during the critical period of respiratory failure, and therefore, may be a good method of life support before removal of plastic casts in patients with PB and in similar situations [22].

In conclusion, PB due to *human adenovirus 7* infection should be considered as a differential diagnosis in children with unalleviated respiratory failure. Flexible bronchoscopy should be performed early to confirm the diagnosis of PB and to clear airway obstructions. Although the disease progresses rapidly, the prognosis of PB caused by *human adenovirus 7* infection was satisfactory with timely and effective treatment.

## Data Availability

The datasets generated and/or analyzed during the current study are available.

## Abbreviations

BALF: Bronchoalveolar lavage fluid
ECMO: Extracorporeal membrane oxygenation
IVIG: Intravenous immunoglobulin
NGS: Next-generation sequencing
PB: Plastic bronchitis
